# 1-Hydroxymethyl-1-methylethan­aminium chloride

**DOI:** 10.1107/S1600536808015018

**Published:** 2008-05-24

**Authors:** Yu-Feng Li, Mei-Li Feng, Shan Liu, Hai-Yu Yang, Hong-Jun Zhu

**Affiliations:** a Department of Applied Chemistry, College of Science, Nanjing University of Technology, Nanjing 210009, People’s Republic of China

## Abstract

The asymmetric unit of the title compound, C_4_H_12_NO^+^·Cl^−^, contains two independent ion pairs. Weak intra­molecular C—H⋯O and N—H⋯O hydrogen bonds result in the formation of three five-membered rings, which have envelope conformations. The crystal structure contains intermolecular O—H⋯Cl, N—H⋯O, N—H⋯Cl and O—H⋯O hydrogen bonds.

## Related literature

For related literature, see: Senkus (1948 ▶). For general background, see: Pazenok (2007 ▶). For bond-length data, see: Allen *et al.* (1987 ▶).
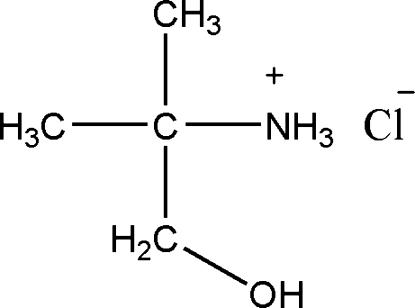


## Experimental

### 

#### Crystal data

C_4_H_12_NO^+^·Cl^−^
*M* *_r_* = 125.60
Monoclinic, 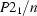

*a* = 6.4940 (13) Å
*b* = 9.5230 (19) Å
*c* = 21.903 (4) Å
β = 91.88 (3)°
*V* = 1353.8 (5) Å^3^
*Z* = 8
Mo *K*α radiation
μ = 0.46 mm^−1^
*T* = 298 (2) K
0.30 × 0.20 × 0.20 mm

#### Data collection

Enraf-Nonius CAD-4 diffractometer
Absorption correction: ψ scan (North *et al.*, 1968 ▶) *T*_min_ = 0.874, *T*_max_ = 0.913
2651 measured reflections
2421 independent reflections
1857 reflections with *I* > 2σ(*I*)
*R*_int_ = 0.020
3 standard reflections frequency: 120 min intensity decay: none

#### Refinement

*R*[*F* ^2^ > 2σ(*F* ^2^)] = 0.040
*wR*(*F* ^2^) = 0.153
*S* = 1.01
2421 reflections
131 parameters
1 restraint
H atoms treated by a mixture of independent and constrained refinement
Δρ_max_ = 0.26 e Å^−3^
Δρ_min_ = −0.28 e Å^−3^

### 

Data collection: *CAD-4 Software* (Enraf–Nonius, 1985 ▶); cell refinement: *CAD-4 Software*; data reduction: *XCAD4* (Harms & Wocadlo, 1995 ▶); program(s) used to solve structure: *SHELXS97* (Sheldrick, 2008 ▶); program(s) used to refine structure: *SHELXL97* (Sheldrick, 2008 ▶); molecular graphics: *SHELXTL* (Sheldrick, 2008 ▶); software used to prepare material for publication: *SHELXTL*.

## Supplementary Material

Crystal structure: contains datablocks I, global. DOI: 10.1107/S1600536808015018/hk2463sup1.cif

Structure factors: contains datablocks I. DOI: 10.1107/S1600536808015018/hk2463Isup2.hkl

Additional supplementary materials:  crystallographic information; 3D view; checkCIF report

## Figures and Tables

**Table 1:** Hydrogen-bond geometry (Å, °)

*D*—H⋯*A*	*D*—H	H⋯*A*	*D*⋯*A*	*D*—H⋯*A*
O1—H1*A*⋯Cl1	0.82 (3)	2.25 (4)	3.067 (2)	174 (3)
N1—H1*B*⋯O2	0.89	2.02	2.876 (3)	161
N1—H1*C*⋯Cl1^i^	0.89	2.33	3.216 (3)	171
N1—H1*G*⋯Cl1^ii^	0.89	2.42	3.261 (2)	157
O2—H2*D*⋯O1^iii^	0.82	1.91	2.731 (3)	175
N2—H2*E*⋯Cl1	0.89	2.53	3.391 (2)	163
N2—H2*E*⋯O2	0.89	2.49	2.811 (3)	102
N2—H2*F*⋯Cl2^iv^	0.89	2.24	3.130 (2)	177
N2—H2*G*⋯Cl2	0.89	2.27	3.144 (2)	169
C2—H2*A*⋯O1	0.96	2.53	2.880 (4)	102
C6—H6*A*⋯O2	0.96	2.59	2.933 (4)	101

Symmetry codes: (i) 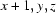; (ii) 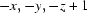; (iii) 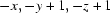; (iv) 
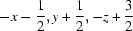
.

**Fig. 1.**
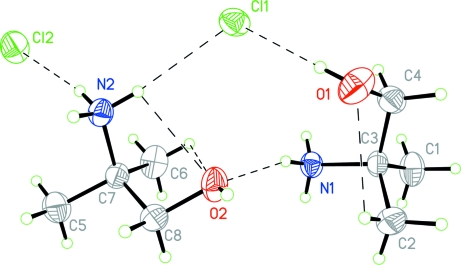
The molecular structure of the title molecule, with the atom-numbering scheme. Displacement ellipsoids are drawn at the 40% probability level. Hydrogen bonds are shown as dashed lines.

**Fig. 2.**
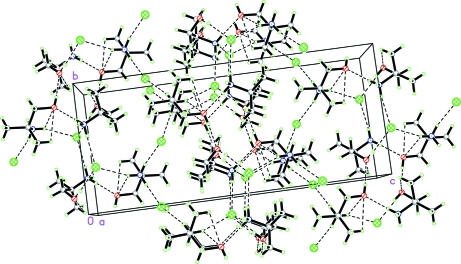
A packing diagram of (I). Hydrogen bonds are shown as dashed lines.

## supplementary crystallographic information

### Comment

The title compound, (I), is an important intermediate for the synthesis of 2-amino-2-methylpropylsulfate, which can be used to synthese 2-methyl-1-(methyl- thio)propane-2-amine (Pazenok, 2007). We report herein the crystal structure of the title compound, (I).

The asymmetric unit of (I) contains two independent molecules (Fig. 1). The bond lengths (Allen *et al.*,  1987) and angles are within normal ranges . The weak intramolecular C-H···O and N-H···O hydrogen bonds (Table 1) result in the formation of three five-membered rings A (C2-C4/O1/H2A), B (C6-C8/O2/H6A) and C (C7/C8/O2/N2/H2E). They adopt envelope conformations, with C3 and C7 atoms displaced by -0.637 (3), -0.686 (4) and 0.711 (3) Å from the planes of the other ring atoms, respectively.

In the crystal structure, intramolecular O-H···Cl, N-H···O and N-H···Cl and intermolecular N-H···Cl and O-H···O hydrogen bonds (Table 1) link the molecules to form a three dimensional network (Fig. 2), in which they may be effective in the stabilization of the structure.

### Experimental

The title compound, (I), was synthesized according to the literature method (Senkus, 1948). Crystals suitable for X-ray analysis were obtained by dissolving (I) (0.30 g, 2.4 mmol) in methanol (25 ml) and evaporating the solvent slowly at room temperature for about 4 d.

### Refinement

H1A atom (for OH) was located in a difference syntheses and refined [O1-H1A = 0.82 (3) Å and U_iso_(H) = 0.070 Å^2^]. The remaining H atoms were positioned geometrically, with O-H = 0.82 Å (for OH), N-H = 0.89 Å (for NH_3_) and C-H = 0.97 and 0.96 Å for methylene and methyl H, respectively, and constrained to ride on their parent atoms with U_iso_(H) = xU_eq_(C,O,N), where x = 1.2 for methylene H, and x = 1.5 for all other H atoms.

### Crystal data

**Table:**

C_4_H_12_NO^+^·Cl^–^	*F*_000_ = 544
*M**_r_* = 125.60	*D*_x_ = 1.232 Mg m^−^^3^
Monoclinic, *P*2_1_/*n*	Mo *K*α radiation λ = 0.71073 Å
Hall symbol: -P 2yn	Cell parameters from 25 reflections
*a* = 6.4940 (13) Å	θ = 11–14º
*b* = 9.5230 (19) Å	µ = 0.46 mm^−^^1^
*c* = 21.903 (4) Å	*T* = 298 (2) K
β = 91.88 (3)º	Block, colorless
*V* = 1353.8 (5) Å^3^	0.30 × 0.20 × 0.20 mm
*Z* = 8	

### Data collection

**Table:**

Enraf-Nonius CAD-4 diffractometer	*R*_int_ = 0.020
Radiation source: fine-focus sealed tube	θ_max_ = 25.2º
Monochromator: graphite	θ_min_ = 1.9º
*T* = 298(2) K	*h* = 0→7
ω/2θ scans	*k* = 0→11
Absorption correction: ψ scan(North *et al.*, 1968)	*l* = −26→26
*T*_min_ = 0.874, *T*_max_ = 0.913	3 standard reflections
2651 measured reflections	every 120 min
2421 independent reflections	intensity decay: none
1857 reflections with *I* > 2σ(*I*)	

### Refinement

**Table:**

Refinement on *F*^2^	Secondary atom site location: difference Fourier map
Least-squares matrix: full	Hydrogen site location: inferred from neighbouring sites
*R*[*F*^2^ > 2σ(*F*^2^)] = 0.040	H atoms treated by a mixture of independent and constrained refinement
*wR*(*F*^2^) = 0.154	*w* = 1/[σ^2^(*F*_o_^2^) + (0.1*P*)^2^ + 0.25*P*] where *P* = (*F*_o_^2^ + 2*F*_c_^2^)/3
*S* = 1.01	(Δ/σ)_max_ = 0.001
2421 reflections	Δρ_max_ = 0.26 e Å^−^^3^
131 parameters	Δρ_min_ = −0.28 e Å^−^^3^
1 restraint	Extinction correction: SHELXL97 (Sheldrick, 2008), Fc^*^=kFc[1+0.001xFc^2^λ^3^/sin(2θ)]^-1/4^
Primary atom site location: structure-invariant direct methods	Extinction coefficient: 0.018 (4)

### Special details

**Table:**

Geometry. All e.s.d.'s (except the e.s.d. in the dihedral angle between two l.s. planes) are estimated using the full covariance matrix. The cell e.s.d.'s are taken into account individually in the estimation of e.s.d.'s in distances, angles and torsion angles; correlations between e.s.d.'s in cell parameters are only used when they are defined by crystal symmetry. An approximate (isotropic) treatment of cell e.s.d.'s is used for estimating e.s.d.'s involving l.s. planes.
Refinement. Refinement of F^2^ against ALL reflections. The weighted R-factor wR and goodness of fit S are based on F^2^, conventional R-factors R are based on F, with F set to zero for negative F^2^. The threshold expression of F^2^ > 2sigma(F^2^) is used only for calculating R-factors(gt) etc. and is not relevant to the choice of reflections for refinement. R-factors based on F^2^ are statistically about twice as large as those based on F, and R- factors based on ALL data will be even larger.

### Fractional atomic coordinates and isotropic or equivalent isotropic displacement parameters (Å^2^)

**Table:**

	*x*	*y*	*z*	*U*_iso_*/*U*_eq_	
Cl1	−0.24225 (12)	0.14202 (8)	0.53408 (3)	0.0414 (3)	
Cl2	−0.24989 (15)	0.01158 (9)	0.76365 (4)	0.0527 (3)	
O1	−0.0723 (3)	0.3564 (2)	0.44418 (11)	0.0468 (6)	
H1A	−0.121 (6)	0.304 (4)	0.4694 (14)	0.070*	
O2	0.1531 (3)	0.3696 (2)	0.58458 (8)	0.0383 (5)	
H2D	0.1269	0.4503	0.5738	0.057*	
N1	0.2905 (4)	0.1957 (2)	0.48659 (10)	0.0313 (6)	
H1B	0.2196	0.2435	0.5138	0.047*	
H1C	0.4221	0.1910	0.4990	0.047*	
H1G	0.2393	0.1093	0.4830	0.047*	
N2	−0.0750 (4)	0.2407 (2)	0.67597 (10)	0.0304 (6)	
H2E	−0.1178	0.2352	0.6370	0.046*	
H2F	−0.1221	0.3195	0.6923	0.046*	
H2G	−0.1221	0.1672	0.6964	0.046*	
C1	0.3962 (6)	0.1829 (4)	0.38056 (15)	0.0485 (9)	
H1D	0.3885	0.2266	0.3411	0.073*	
H1E	0.3397	0.0899	0.3777	0.073*	
H1F	0.5376	0.1778	0.3947	0.073*	
C2	0.3599 (5)	0.4154 (3)	0.43319 (14)	0.0410 (7)	
H2A	0.2787	0.4665	0.4615	0.061*	
H2B	0.3559	0.4628	0.3945	0.061*	
H2C	0.4998	0.4101	0.4486	0.061*	
C3	0.2741 (4)	0.2692 (3)	0.42531 (12)	0.0300 (6)	
C4	0.0495 (5)	0.2723 (3)	0.40505 (14)	0.0383 (7)	
H4A	0.0375	0.3090	0.3638	0.046*	
H4B	−0.0039	0.1771	0.4044	0.046*	
C5	0.2240 (5)	0.2504 (4)	0.74660 (14)	0.0467 (8)	
H5A	0.1744	0.3366	0.7635	0.070*	
H5B	0.3717	0.2483	0.7502	0.070*	
H5C	0.1685	0.1724	0.7684	0.070*	
C6	0.2298 (5)	0.1056 (3)	0.65041 (15)	0.0464 (8)	
H6A	0.1841	0.1034	0.6083	0.070*	
H6B	0.1740	0.0265	0.6714	0.070*	
H6C	0.3776	0.1016	0.6530	0.070*	
C7	0.1566 (4)	0.2409 (3)	0.67977 (12)	0.0305 (6)	
C8	0.2295 (5)	0.3699 (3)	0.64625 (13)	0.0369 (7)	
H8A	0.1820	0.4536	0.6667	0.044*	
H8B	0.3789	0.3717	0.6470	0.044*	

### Atomic displacement parameters (Å^2^)

**Table:**

	*U*^11^	*U*^22^	*U*^33^	*U*^12^	*U*^13^	*U*^23^
Cl1	0.0418 (5)	0.0385 (5)	0.0435 (5)	−0.0061 (3)	−0.0065 (3)	0.0059 (3)
Cl2	0.0708 (6)	0.0471 (5)	0.0406 (5)	−0.0228 (4)	0.0074 (4)	0.0050 (3)
O1	0.0390 (12)	0.0394 (13)	0.0628 (15)	0.0087 (10)	0.0117 (11)	0.0180 (10)
O2	0.0545 (13)	0.0310 (11)	0.0296 (11)	0.0003 (10)	0.0050 (9)	0.0044 (8)
N1	0.0321 (13)	0.0338 (12)	0.0281 (12)	0.0047 (10)	0.0007 (10)	0.0042 (10)
N2	0.0340 (13)	0.0274 (12)	0.0299 (12)	0.0009 (10)	0.0042 (10)	−0.0003 (9)
C1	0.059 (2)	0.053 (2)	0.0340 (16)	0.0105 (17)	0.0127 (15)	−0.0028 (14)
C2	0.0424 (18)	0.0367 (17)	0.0435 (17)	−0.0089 (14)	−0.0020 (14)	0.0048 (13)
C3	0.0355 (15)	0.0314 (15)	0.0230 (13)	−0.0019 (12)	0.0018 (11)	0.0026 (11)
C4	0.0381 (17)	0.0379 (16)	0.0385 (16)	−0.0033 (14)	−0.0065 (13)	0.0047 (13)
C5	0.052 (2)	0.057 (2)	0.0313 (16)	−0.0046 (17)	−0.0061 (14)	0.0075 (14)
C6	0.056 (2)	0.0366 (17)	0.0473 (19)	0.0154 (15)	0.0069 (16)	0.0066 (14)
C7	0.0299 (14)	0.0332 (15)	0.0284 (14)	0.0004 (12)	0.0029 (11)	0.0015 (11)
C8	0.0416 (17)	0.0365 (16)	0.0328 (15)	−0.0097 (14)	0.0020 (13)	0.0024 (12)

### Geometric parameters (Å, °)

**Table:**

O1—C4	1.430 (4)	O2—C8	1.424 (3)
O1—H1A	0.82 (3)	O2—H2D	0.8200
N1—C3	1.515 (3)	N2—C7	1.503 (4)
N1—H1B	0.8900	N2—H2E	0.8900
N1—H1C	0.8900	N2—H2F	0.8900
N1—H1G	0.8900	N2—H2G	0.8900
C1—C3	1.522 (4)	C5—C7	1.517 (4)
C1—H1D	0.9600	C5—H5A	0.9600
C1—H1E	0.9600	C5—H5B	0.9600
C1—H1F	0.9600	C5—H5C	0.9600
C2—C3	1.507 (4)	C6—C7	1.523 (4)
C2—H2A	0.9600	C6—H6A	0.9600
C2—H2B	0.9600	C6—H6B	0.9600
C2—H2C	0.9600	C6—H6C	0.9600
C3—C4	1.511 (4)	C7—C8	1.515 (4)
C4—H4A	0.9700	C8—H8A	0.9700
C4—H4B	0.9700	C8—H8B	0.9700
			
C4—O1—H1A	107 (3)	C8—O2—H2D	109.5
C3—N1—H1B	109.5	C7—N2—H2E	109.5
C3—N1—H1C	109.5	C7—N2—H2F	109.5
H1B—N1—H1C	109.5	H2E—N2—H2F	109.5
C3—N1—H1G	109.5	C7—N2—H2G	109.5
H1B—N1—H1G	109.5	H2E—N2—H2G	109.5
H1C—N1—H1G	109.5	H2F—N2—H2G	109.5
C3—C1—H1D	109.5	C7—C5—H5A	109.5
C3—C1—H1E	109.5	C7—C5—H5B	109.5
H1D—C1—H1E	109.5	H5A—C5—H5B	109.5
C3—C1—H1F	109.5	C7—C5—H5C	109.5
H1D—C1—H1F	109.5	H5A—C5—H5C	109.5
H1E—C1—H1F	109.5	H5B—C5—H5C	109.5
C3—C2—H2A	109.5	C7—C6—H6A	109.5
C3—C2—H2B	109.5	C7—C6—H6B	109.5
H2A—C2—H2B	109.5	H6A—C6—H6B	109.5
C3—C2—H2C	109.5	C7—C6—H6C	109.5
H2A—C2—H2C	109.5	H6A—C6—H6C	109.5
H2B—C2—H2C	109.5	H6B—C6—H6C	109.5
C2—C3—C4	111.4 (2)	N2—C7—C8	107.6 (2)
C2—C3—N1	108.1 (2)	N2—C7—C5	108.1 (2)
C4—C3—N1	107.9 (2)	C8—C7—C5	109.5 (2)
C2—C3—C1	112.0 (3)	N2—C7—C6	107.5 (2)
C4—C3—C1	109.9 (3)	C8—C7—C6	112.0 (2)
N1—C3—C1	107.3 (2)	C5—C7—C6	112.0 (2)
O1—C4—C3	112.5 (2)	O2—C8—C7	110.7 (2)
O1—C4—H4A	109.1	O2—C8—H8A	109.5
C3—C4—H4A	109.1	C7—C8—H8A	109.5
O1—C4—H4B	109.1	O2—C8—H8B	109.5
C3—C4—H4B	109.1	C7—C8—H8B	109.5
H4A—C4—H4B	107.8	H8A—C8—H8B	108.1
			
C2—C3—C4—O1	−53.1 (3)	N2—C7—C8—O2	−57.7 (3)
N1—C3—C4—O1	65.4 (3)	C5—C7—C8—O2	−174.9 (2)
C1—C3—C4—O1	−177.8 (2)	C6—C7—C8—O2	60.2 (3)

### Hydrogen-bond geometry (Å, °)

**Table:**

*D*—H···*A*	*D*—H	H···*A*	*D*···*A*	*D*—H···*A*
O1—H1A···Cl1	0.82 (3)	2.25 (4)	3.067 (2)	174 (3)
N1—H1B···O2	0.89	2.02	2.876 (3)	161
N1—H1C···Cl1^i^	0.89	2.33	3.216 (3)	171
N1—H1G···Cl1^ii^	0.89	2.42	3.261 (2)	157
O2—H2D···O1^iii^	0.82	1.91	2.731 (3)	175
N2—H2E···Cl1	0.89	2.53	3.391 (2)	163
N2—H2E···O2	0.89	2.49	2.811 (3)	102
N2—H2F···Cl2^iv^	0.89	2.24	3.130 (2)	177
N2—H2G···Cl2	0.89	2.27	3.144 (2)	169
C2—H2A···O1	0.96	2.53	2.880 (4)	102
C6—H6A···O2	0.96	2.59	2.933 (4)	101

Symmetry codes: (i) *x*+1, *y*, *z*; (ii) −*x*, −*y*, −*z*+1; (iii) −*x*, −*y*+1, −*z*+1; (iv) −*x*−1/2, *y*+1/2, −*z*+3/2.
